# Myosteatosis is associated with adverse in-hospital outcomes and CONUT score in hospitalized internal medicine patients

**DOI:** 10.1007/s00592-026-02785-3

**Published:** 2026-08-31

**Authors:** Nicoletta Miano, Maurizio Di Marco, Sabrina Scilletta, Giorgia Todaro, Giosiana Bosco, Francesco Di Giacomo Barbagallo, Roberto Scicali, Salvatore Piro, Antonino Di Pino

**Affiliations:** 1 Department of Clinical and Experimental Medicine, University of Catania, Via Palermo 636, 95122 Catania, Italy; 2 Department of Medicine and Surgery, “Kore” University of Enna, Enna, 94100 Italy

**Keywords:** Myosteatosis, Internal medicine, Nutritional status, CONUT score, In-hospital death, Diabetes

## Abstract

**Aims:**

Myosteatosis is a marker of malnutrition and of adverse clinical outcomes and diabetes is among its contributors. Currently, no single universally accepted method exists to screen for malnutrition, but COntrolling NUTritional status (CONUT) score seems to be promising. This study aimed to assess the impact of myosteatosis on in-hospital outcomes and to test its association with CONUT score in people with and without type 2 diabetes.

**Methods:**

227 patients aged > 40 years admitted to an Internal Medicine Department were consecutively recruited, recording in-hospital outcomes. Myosteatosis was assessed as multifidus muscle to subcutaneous fat (MM/F) attenuation ratio at computed-tomography scan. CONUT was calculated using albumin, lymphocyte count and total cholesterol. The population was stratified, based on sex-differentiated tertiles of MM/F, in a low myosteatosis group (*n* = 152) and a high myosteatosis group (*n* = 75; top tertile of MM/F).

**Results:**

The high myosteatosis group showed a higher prevalence of in-hospital death (12.5 vs. 26%, *P* < 0.001) and pneumonia. Moreover, the high myosteatosis group experienced a longer length-of-stay and was more likely to have cultures positive for multi-drug-resistant germs. In-hospital death was independently associated with MM/F ratio (OR 2.74, 95%CI 1.11–7.75, *P* = 0.035). Diabetes did not show significant interaction on this association (*P*-for-interaction = 0.43). From multiple regression model, MM/F was independently associated with CONUT (β = 0.30, *P* < 0.001).

**Conclusions:**

Myosteatosis was associated with adverse in-hospital outcomes. CONUT score was independently associated with the imaging-derived MM/F ratio. Further studies specifically designed to evaluate diagnostic performance are required before CONUT can be proposed as a screening tool for myosteatosis.

**Supplementary Information:**

The online version contains supplementary material available at https://doi.org/10.1007/s00592-026-02785-3.

## Introduction

Malnutrition is a condition resulting from inadequate nutritional intake or impaired nutrient absorption, leading to altered body composition, diminished physical and cognitive function, and ultimately adverse clinical outcomes [1, 2]. Depending on the population studied and the diagnostic criteria applied, the worldwide prevalence of disease-related malnutrition ranges from 20% to 50% [3]. In older adults, malnutrition frequently coexists with sarcopenia, and their concomitant presence is associated with unfavourable clinical outcomes, including falls, disability, prolonged hospitalization, institutionalization, and increased mortality [4]. Despite its clinical relevance, there is currently no single universally accepted gold standard method for the diagnosis of malnutrition, and existing tools are often inadequate for routine clinical practice [1].

Closely related to the concepts of malnutrition and sarcopenia is myosteatosis, a relatively novel term describing impaired skeletal muscle quality due to excessive intramuscular adipose tissue infiltration [5]. Myosteatosis has gained increasing attention as a potential marker of sarcopenia, as fat infiltration within muscle may compromise muscle strength and mobility and increase morbidity risk, particularly in older adults [6–8]. Although more prevalent among older and obese individuals, myosteatosis can also occur in individuals with normal body weight [6]. It represents a chronic inflammatory condition associated with reduced muscle function, metabolic derangements, and decreased survival [9]. Furthermore, myosteatosis may contribute to hyperglycaemia, insulin resistance, and dyslipidemia through multiple mechanisms, independently of overall obesity and visceral adiposity [10, 11]. People with diabetes have been shown to have higher intra- and extramyocellular lipid content [12], and ectopic fat distribution may predict the risk of type 2 diabetes [13]. This bidirectional link may be partially explained by the roles of insulin resistance and low grade inflammation [14, 15]. The diagnostic assessment of myosteatosis relies primarily on imaging-based techniques, particularly computed tomography (CT) and magnetic resonance imaging (MRI), which allow quantification of intramuscular fat infiltration [7]. Other modalities, such as ultrasound, are emerging but remain under investigation [5, 8].

In parallel, growing interest has focused on the COntrolling NUTritional status (CONUT) score, a recently introduced nutritional-risk screening index, that has also shown potential prognostic value across various clinical contexts [16]. The CONUT score is based on total peripheral lymphocyte count, serum albumin concentration, and total cholesterol levels. Thus, this index allows for the screening of patients at risk of poor nutritional status using very simple biochemical measures, without requiring the patients’ cooperation. The use of the CONUT score in clinical studies is increasing, even though several other screening indexes have been proposed, including the Nutritional Risk Screening 2002 and the Mini Nutritional Assessment – Short Form [17, 18]. Furthermore, although a comprehensive diagnosis of malnutrition requires more advanced protocols such as the Global Leadership Initiative on Malnutrition criteria [19], recent studies of our group have shown that CONUT Score may be used as simple index for nutritional screening to identify patients at risk of adverse outcomes [20, 21].

The aims of the present study were to evaluate the association of myosteatosis on in-hospital outcomes in patients admitted to an Internal Medicine Department – with a primary focus on all-cause mortality – and to investigate the association between myosteatosis and the CONUT score.

## Materials & methods

### Patients

We consecutively recruited patients who were admitted to the Internal Medicine Department of the Azienda Ospedaliera di Alta Specializzazione Garibaldi Nesima, Catania, Italy from September to December 2023. Inclusion criteria were age > 40 years and undergoing an abdomen CT scan for clinical management. We collected the following data: (1) age, gender, comorbidities; (2) clinical events occurred during hospitalization like death, days of in-hospital stay, diagnosis of sepsis (at admission or during the hospitalization), and pneumonia; (3) clinical and biochemical characteristics at the moment of hospitalization as previously described [22], such as systolic and diastolic blood pressure, glycaemia, creatinine, estimated glomerular filtration rate, total cholesterol, high density lipoprotein cholesterol (HDL-c), low density lipoprotein cholesterol (LDL-c), triglycerides, total proteins, albumin, alanine aminotransferase, aspartate aminotransferase, N-terminal pro brain natriuretic peptide, procalcitonin, high sensitivity C-reactive protein (hs-CRP), complete blood count, haemoglobin, haematocrit, and international normalized ratio.

### Evaluation of myosteatosis

Skeletal muscle density was assessed based on X-ray attenuation in Hounsfield Units on CT-scan, in agreement with recent literature. One 60-mm^2^ circular region of interest (ROI) was placed on each multifidus muscle (right and left) and five ROIs were placed on the subcutaneous fat at the level of the navel. The mean value of the two multifidus muscle measurements and the mean value of the five subcutaneous fat measurements were then calculated. Finally, the ratio between these two means was calculated, yielding the multifidus muscle-to-subcutaneous fat ratio (MM/F ratio), an index of the intramuscular fat content [23]. A higher MM/F ratio indicates a higher intramuscular fat content.

### Study groups

Participants were stratified according to MM/F ratio in two groups: low myosteatosis group (MM/F in the lower and intermediate tertile) and high myosteatosis group (MM/F in top tertile).

### Calculations

According to the original study [16], the serum albumin concentration, total peripheral lymphocyte count, and total cholesterol concentration were used to calculate the CONUT score. The measures were performed at the time of admission to prevent the influence of major therapeutic interventions. Albumin concentrations ≥ 3.5 g/dL, 3.0–3.49 g/dL, 2.5–2.99 g/dL, and ≤ 2.5 g/dL were scored as 0, 2, 4, 6, respectively. Total lymphocyte counts ≥ 1600/mm^3^, 1200–1599/mm^3^, 800–1199/mm^3^, and ≤ 800/mm^3^ were scored as 0, 1, 2, 3, respectively. Total cholesterol concentrations ≥ 180 mg/dL, 140–179 mg/dL, 100–139 mg/dL, and ≤ 100 mg/dL were scored as 0, 1, 2, 3, respectively. The CONUT score was calculated as the sum of the three component scores. The Prognostic Nutritional Index (PNI) was calculated following the original work [24]. The Chronic Kidney Disease Epidemiology Collaboration (CKD-EPI) equation was used to calculate the estimated glomerular filtration rate (eGFR) [25].

### Statistical analysis

Clinical variable and biomedical measures were compared, and graphics were done through R software (version 4.5.2 for Windows, R Core Team). Data is given as mean ± SD or median (1st, 3rd quartile). Each variable’s distributional characteristics including normality were evaluated by the Kolmogorov-Smirnov test. Comparisons between the 2 groups were performed using t test for parametric continuous variables, Kruskal-Wallis test for non-parametric continuous variables, and the Chi-square test for non-continuous variables. A *P* value less than 0.05 was considered statistically significant. When necessary, numerical variables were logarithmically transformed to reduce skewness, and values are expressed as median and interquartile range.

The primary outcome measure of the present study was the risk of all-cause death during the index hospitalization according to the MM/F ratio. The secondary outcomes included days of in-hospital stays, the diagnosis of sepsis and pneumonia during hospitalization, culture isolation of multi-drug-resistant (MDR) germs, acute kidney injury, and blood transfusions. We performed logistic regression analyses to assess the association between in-hospital death and MM/F ratio. We fitted two models: the first model was adjusted for age and sex; whereas the second model was additionally adjusted for covariates that could impact on myosteatosis and/or in-hospital mortality, particularly history of type 2 diabetes, eGFR (capturing both chronic and acute kidney dysfunction), active malignancies, and sepsis during the hospitalization. As sensitivity analysis, the model was refitted excluding sepsis because of the potential risk of collider bias. Furthermore, the results of the fully adjusted model were replicated using penalized Firth’s logistic regression., Moreover, the interaction between type 2 diabetes and the association between MM/F and in-hospital mortality was tested. Finally, a multiple linear regression analysis was performed to assess the major determinants of MM/F ratio. In this model, the MM/F ratio was the dependent variable, while age, sex, bedridden status, comorbidities (active malignancies, history of type 2 diabetes, coronary heart disease [CHD], stroke/transient ischemic attack [TIA], chronic kidney disease [CKD], dementia, and chronic obstructive pulmonary disease [COPD]), and CONUT score were included as explanatory variables. As a sensitivity analysis, the model was also refitted including hs-CRP, as the components of the CONUT score components may be influenced by inflammation.

## Results

### General characteristics of the study population

According to the eligibility criteria, 227 individuals were recruited.

Based on sex-differentiated tertiles of the MM/F ratio, participants were divided into a low myosteatosis group (*n* = 152; MM/F < 0.255 for females and < − 0.094 for males) and a high myosteatosis group (*n* = 75; MM/F in the top tertile, > 0.255 for females and > − 0.094 for males).

As shown in Table 1, individuals in the high myosteatosis group were older and more frequently bedridden than those in the MM/F low group. Regarding comorbidities, participants in the high myosteatosis group had a higher prevalence of CHD and dementia, with a trend toward a higher prevalence of CKD. In contrast, the prevalence of history of stroke/TIA, COPD, type 2 diabetes, and active malignancies was homogeneous in the study population. The high myosteatosis group showed also lower eGFR and haemoglobin levels compared with the low myosteatosis group.

**Table 1:** Clinical characteristics of the study population

Age, years	Overall population (*n* = 227)	Low myosteatosis (*n* = 152)	High myosteatosis (*n* = 75)	*P* value
	72.33 ± 15.17	69.07 ± 16.10	78.95 ± 10.38	< 0.001
Sex, no. (%) of males	128 (56.4)	86 (56.6)	42 (56.0)	1.00
MM/F ratio	-0.08 ± 0.45	-0.31 ± 0.26	0.37 ± 0.41	**< 0.001**
CONUT score	5.53 ± 2.77	4.87 ± 2.67	6.79 ± 2.53	**< 0.001**
PNI	38.25 ± 9.41	40.28 ± 10.23	34.20 ± 5.65	**< 0.001**
Being bedridden, no. (%)	81 (35.7)	34 (22.4)	47 (62.7)	**< 0.001**
History of CHD, no. (%)	40 (17.6)	21 (13.8)	19 (25.3)	**0.032**
History of TIA or stroke, no. (%)	13 (5.7)	8 (5.3)	5 (6.7)	0.18
History of dementia, no. (%)	46 (20.3)	22 (14.5)	24 (32.0)	**0.0020**
History of COPD, no. (%)	63 (27.7)	38 (25.0)	25 (33.3)	0.19
History of liver disease, no. (%)	21 (9.2)	11 (7.2)	10 (13.3)	0.14
History of type 2 diabetes, no. (%)	68 (30.0)	42 (27.6)	26 (34.5)	0.28
History of CKD, no. (%)	54 (23.8)	31 (20.4)	23 (30.7)	0.090
Active malignancies, no. (%)	78 (34.3)	49 (32.2)	29 (38.7)	0.34
SBP, mmHg	125.19 ± 19.60	125.33 ± 18.66	125.02 ± 20.86	0.93
DBP, mmHg	70.64 ± 10.58	71.17 ± 11.36	70.00 ± 9.64	0.57
Urea, mg/dl	49.00 [31.00, 81.00]	46.00 [30.00, 74.75]	54.00 [33.00, 88.0]	0.29
eGFR, ml/min/1,73m^2^	68.35 ± 32.95	71.56 ± 34.11	61.94 ± 29.69	**0.039**
Sodium, mmol/l	138.62 ± 4.51	138.38 ± 3.58	138.90 ± 5.44	0.55
Potassium, mmol/l	3.81 ± 0.53	3.96 ± 0.50	3.63 ± 0.53	**0.001**
Fasting glucose, mg/dl	109.62 ± 49.32	108.86 ± 46.54	111.08 ± 54.59	0.75
Total cholesterol, mg/dl	145.71 ± 53.19	149.56 ± 53.71	138.22 ± 51.69	0.14
HDL-c, mg/dl	31.21 ± 14.75	31.70 ± 14.62	30.16 ± 15.12	0.49
LDL-c, mg/dl	88.97 ± 48.01	92.61 ± 47.87	81.15 ± 47.75	0.12
Triglycerides, mg/dl	115.00 [85.00, 154.00]	116.00 [82.00, 156.00]	112.50 [85.50, 151.00]	0.93
Total protein, g/dl	5.83 ± 0.83	5.94 ± 0.88	5.61 ± 0.68	**0.005**
Albumin, g/dl	3.06 ± 0.59	3.18 ± 0.62	2.83 ± 0.42	**< 0.001**
AST, U/l	23.00 [17.00, 36.75]	23.50 [17.75, 38.25]	22.00 [16.25, 34.50]	0.35
ALT, U/l	15.00 [8.00, 32.50]	20.00 [12.00, 37.25]	10.00 [6.00, 20.75]	**0.001**
Total bilirubin, mg/dl	0.80 [0.50, 1.20]	0.70 [0.50, 1.12]	0.80 [0.50, 1.20]	0.53
γ-GT, U/l	31.00 [18.00, 54.00]	33.00 [18.50, 56.50]	27.00 [18.00, 46.00]	0.71
ALP, U/l	72.00 [59.00, 100.50]	72.50 [59.25, 103.75]	71.00 [59.00, 89.00]	0.80
hs-CRP, mg/dL	6.12 [1.92, 12.53]	5.89 [1.32, 12.08]	6.13 [3.14, 12.65]	0.36
Procalcitonin, µg/l	0.16 [0.05, 0.69]	0.13 [0.05, 0.49]	0.25 [0.06, 1.86]	0.054
WBC, 10^3/µl	10.12 ± 7.26	10.47 ± 7.45	9.42 ± 6.86	0.31
Neutrophils count, 10^3/µl	7.58 ± 6.35	7.64 ± 6.10	7.45 ± 6.85	0.83
Lymphocytes count, 10^3/µl	1.53 ± 1.34	1.70 ± 1.54	1.18 ± 0.66	**0.006**
RBC, 10^6/ µl	3.82 ± 0.81	3.88 ± 0.82	3.71 ± 0.76	0.14
Hemoglobin, g/dl	11.01 ± 2.28	11.25 ± 2.41	10.55 ± 1.94	**0.032**
Platelet, 10^3/µl	236.11 ± 120.21	244.42 ± 127.15	219.48 ± 103.78	0.14
Serum iron, µg/dl	55.43 ± 38.82	54.48 ± 35.02	57.29 ± 45.55	0.61
Ferritin, ng/ml	305.00 [135.25, 657.00]	299.00 [134.50, 579.50]	325.00 [148.50, 703.50]	0.43
Folate, ng/ml	4.75 [3.00, 11.95]	4.80 [2.90, 12.50]	4.70 [3.05, 8.05]	0.83
B12 vitamin, pg/ml	470.50 [265.25, 808.25]	460.00 [237.00, 815.50]	494.00 [299.50, 731.50]	0.34
Fibrinogen, mg/dl	489.96 ± 219.49	500.69 ± 231.38	468.79 ± 193.63	0.31
INR	1.32 ± 0.41	1.36 ± 0.53	1.28 ± 0.16	0.35
PTT, sec	37.15 ± 14.55	37.48 ± 16.07	36.76 ± 12.65	0.80
NT-proBNP, pg/ml	168.70 [80.40, 565.00]	187.60 [102.25, 567.50]	154.70 [71.78, 357.02]	0.75

Data are presented as mean ± SD, median [1st, 3rd quartile], or percentage and compared with t-test, Mann-Whitney, or χ^2^ as appropriate. Participants are split according to multifidus muscle / subcutaneous fat (MM/F) sex-differentiated tertiles, including individuals in the top tertile in the MM/F high group. The cut-offs were 0.255 for females and − 0.094 for males. CONUT: controlling nutritional status; CHD: Coronary heart disease; TIA: transient ischemic attack; COPD: chronic obstructive pulmonary disease; CKD: chronic kidney disease; SBP: systolic blood pressure; DBP: diastolic blood pressure; eGFR: estimated glomerular filtration rate; HDL-c: high-density lipoprotein cholesterol; LDL-c: low-density lipoprotein cholesterol, AST: aspartate aminotransferase; ALT: alanine aminotransferase; γ-GT: gamma-glutamyl-transpeptidase; hs-CRP: high-sensitivity C-reactive protein; WBC: white blood cell count; RBC: red blood cell count; INR: international normalized ratio; PTT: partial thromboplastin time; NT-proBNP: N-terminal pro brain natriuretic peptide

With respect to indexes indicating risk of poor nutritional status, individuals in the high myosteatosis group had higher CONUT scores and lower PNI values than those in the low myosteatosis group. Moreover, we found that participants in the high myosteatosis group had lower levels of albumin, total proteins and lymphocyte count.

### In-hospital outcomes in the study population

As shown in Table 2, participants in the high myosteatosis group had a higher prevalence of in-hospital death and pneumonia compared with those in the low myosteatosis group. Particularly, 10/39 (25.6%) death were in participants with type 2 diabetes, and 29/39 (74.4%) in those without it. Moreover, the high myosteatosis group experienced a longer length of hospital stay and were more likely to have cultures positive for MDR germs, with a trend toward higher incidence of sepsis.

**Table 2:** In-hospital outcomes of the study population

	Overall population (*n* = 227)	MM/F low (*n* = 152)	MM/F high (*n* = 75)	*P* value
In-hospital death, no. (%)	39 (17.2)	19 (12.5)	20 (26.7)	< 0.001
Length of hospital stay, days	16.05 ± 13.48	14.34 ± 10.28	19.04 ± 17.43	**0.017**
Sepsis, no. (%)	86 (37.9)	52 (34.2)	34 (45.3)	0.10
Culture isolation of MDR germs, no. (%)	39 (17.2)	18 (11.8)	21 (28.0)	**0.0024**
Pneumonia, no. (%)	65 (28.6)	28 (18.4)	37 (49.3)	**< 0.001**
Acute kidney injury, no. (%)	31 (13.6)	18 (11.8)	13 (17.3)	0.26
Blood transfusions, no. (%)	45 (19.8)	30 (19.7)	15 (20.0)	1.00

Data are presented as mean ± SD or absolute number (percentage) and compared with t-test or χ^2^ as appropriate. Participants are split according to multifidus muscle / subcutaneous fat (MM/F) sex-specific tertiles, including individuals in the top tertile in the MM/F high group. The cut-offs were 0.255 for females and − 0.094 for males. MDR: multidrug resistant

### Logistic regression analysis to assess the association of in-hospital death with MM/F ratio

**Fig. 1:**
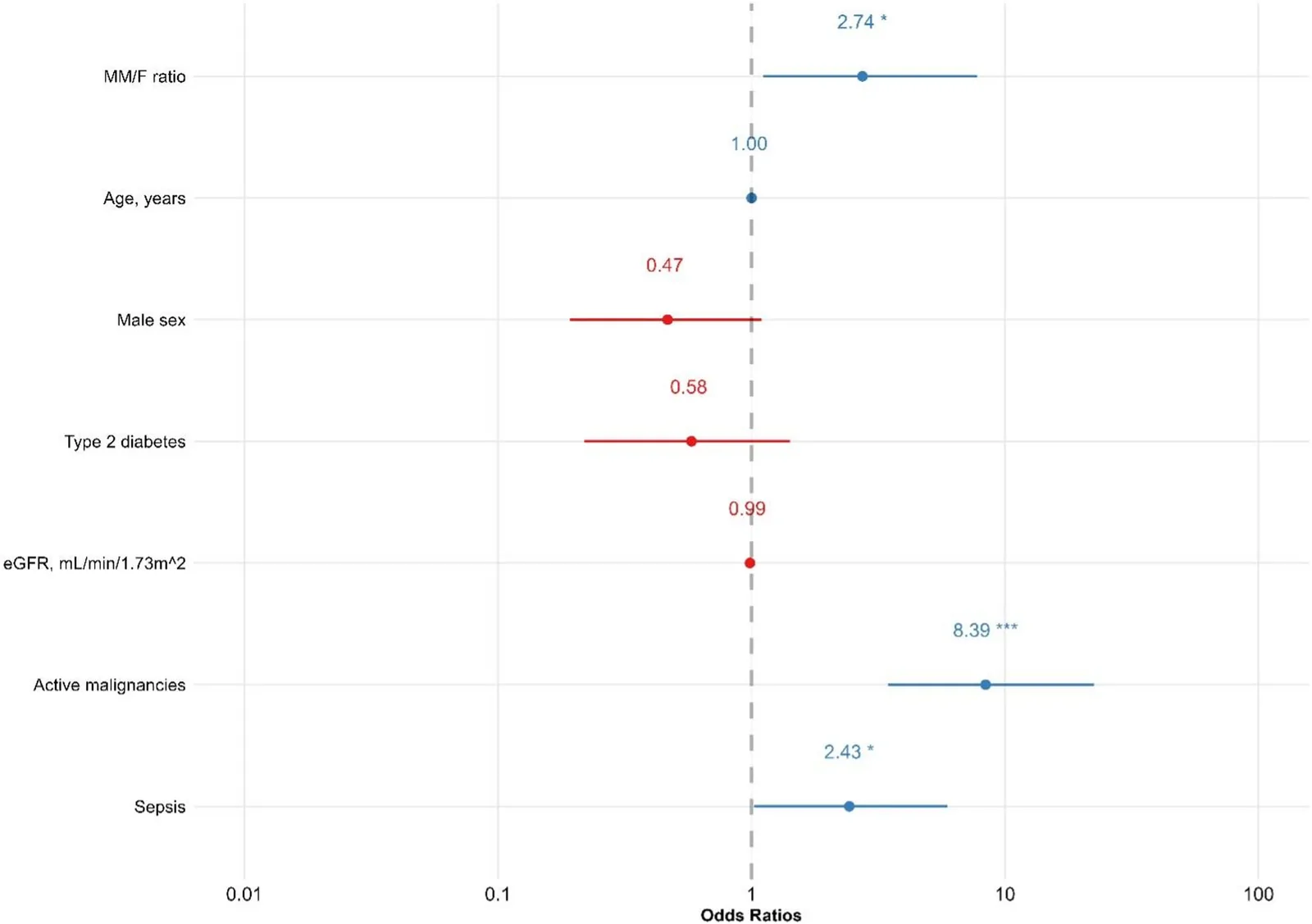
Forest plot of the logistic regression analysis assessing the association of in-hospital death with MM/F ratio. The forest plot shows the odds ratios (OR) and the 95% confidence intervals of the logistic regression model with in-hospital death as dependent variable and multifidus muscle to subcutaneous fat (MM/F) ratio, age, sex, history of type 2 diabetes, estimated glomerular filtration rate (eGFR),, active malignancies, and sepsis during in-hospital stay as explanatory variables. *P < 0.05, ***P < 0.001. Red: OR < 1.00; blue: OR ≥ 1.00

After adjusting for age and sex (Supplementary Table S1), in-hospital death was independently associated with MM/F ratio (odds ratio [OR] 2.45, 95% confidence interval [CI] 1.07–6.21, *P* = 0.040). Furthermore, after further adjustment for history of type 2 diabetes, eGFR, presence of active malignancies, and sepsis during in-hospital stay (Supplementary Table S1 and Fig. 1), the association remained significant (OR 2.74, 95%CI 1.11–7.75, *P* = 0.035).

### Sensitivity analyses to assess the robustness of the association between in-hospital death and MM/F ratio

When sepsis during in-hospital stay was excluded from the logistic regression model, the estimate of the association between in-hospital death and MM/F ratio remained consistent (OR 2.73, 95%CI 1.10–7.82, *P* = 0.043). Moreover, when the full model was refitted as penalized Firth’s logistic regression (Supplementary Table S2) in-hospital death was also significantly associated with MM/F ratio (OR 2.49, 95%CI 1.04–5.96, *P* = 0.032).

### Assessment of the interaction of diabetes on in-hospital death with MM/F ratio

When type 2 diabetes was treated as interaction term in the logistic regression analysis, it did not show significant interaction on the association between in-hospital death and MM/F ratio (coefficient − 0.91, 95%CI -3.2–1.41, *P* for interaction = 0.43) and therefore no statistically significant differences between subgroups were detected.

### Multiple regression analysis to assess the major determinants of MM/F ratio

**Fig. 2:**
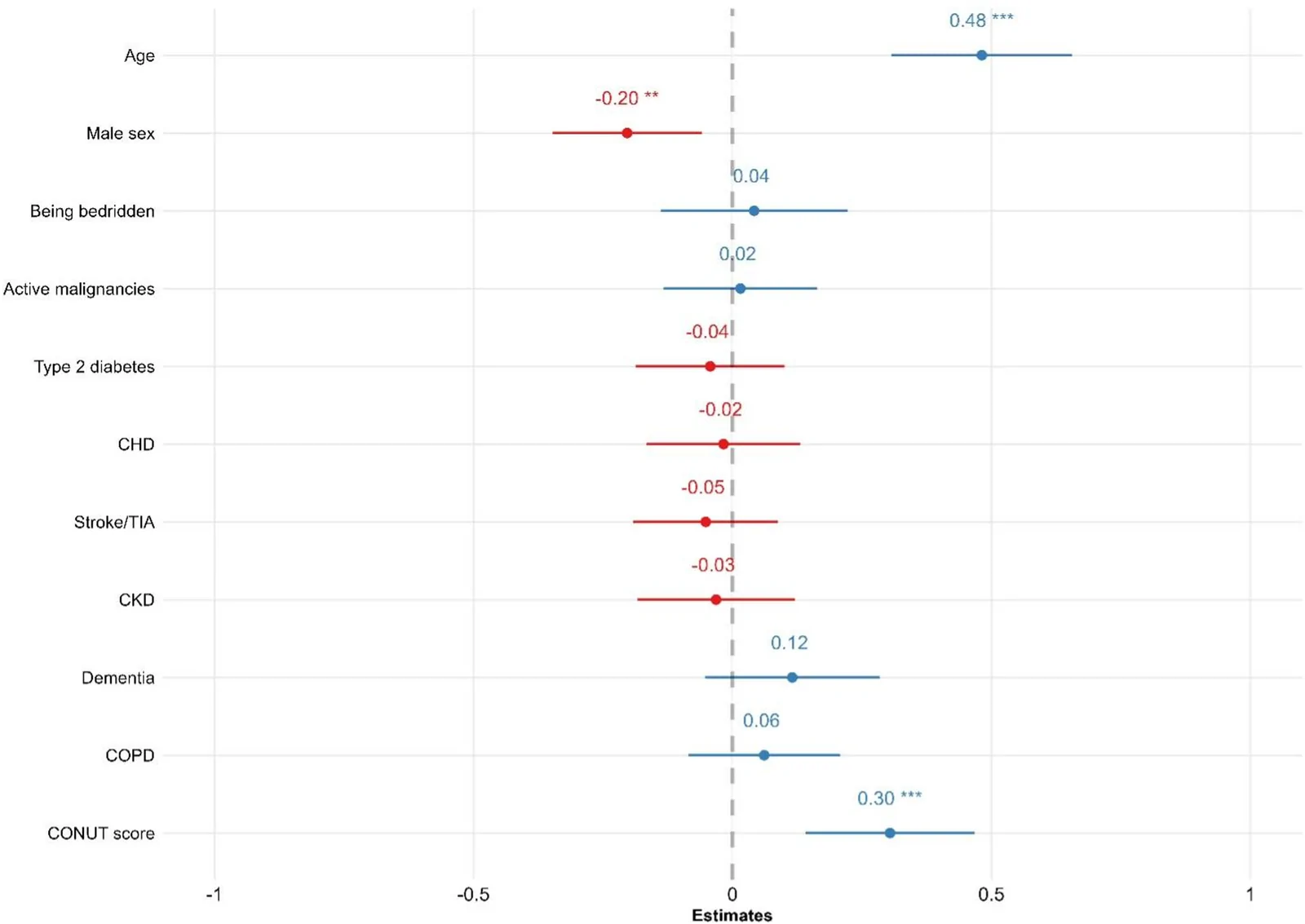
Forest plot of the multiple regression analysis assessing the major determinants of MM/F ratio. The forest plot shows the standardized β coefficients and the 95% confidence intervals of the multiple regression model with multifidus muscle to subcutaneous fat (MM/F) ratio as dependent variable and age, sex, being bedridden, active malignancies, history of diabetes, coronary heart disease (CHD), stroke/transient ischemic attack (TIA), chronic kidney disease (CKD), dementia, chronic obstructive pulmonary disease (COPD) and controlling nutritional status (CONUT) score as explanatory variables. **P < 0.01, *** *P* < 0.001. Blue: β ≥ 1.00, red: β < 1.00

After multiple regression analysis adjusted for age, sex, being bedridden, comorbidities (malignancies, history of diabetes, CHD, stroke/TIA, CKD, dementia, and COPD), and CONUT (Supplementary table S3 and Fig. 2), MM/F ratio was independently associated with age (β = 0.48, 95%CI 0.31–0.66, *P* < 0.001) and inversely with male sex (β = -0.20, 95%CI -0.35 – -0.06, *P* = 0.0061). Furthermore, MM/F ratio was directly associated with CONUT score (β = 0.30, 95%CI 0.14–0.47, *P* < 0.001).

After including hs-CRP among confounders, the association between MM/F ratio and CONUT score remained significant (β = 0.33, 95%CI 0.15–0.51, *P* < 0.001).

## Discussion

In this study, we investigated the association between myosteatosis—defined by an elevated muscle-to-subcutaneous fat (MM/F) ratio assessed through CT scan—and adverse in-hospital outcomes. Additionally, we examined the relationship between myosteatosis and CONUT score, a recently introduced screening index to identify individuals at risk of poor nutritional status. This is the first study aiming to evaluate the prognostic association of myosteatosis with adverse in-hospital outcomes in an Internal Medicine Department and to correlate CONUT score to myosteatosis. We found that a higher MM/F ratio at admission, indicating higher muscle fat content, was significantly associated with in-hospital mortality even after adjusting for confounders. No statistically significant effect modification by type 2 diabetes was detected, although the study may have had limited power to identify subgroup differences. Moreover, the CONUT score was independently associated with MM/F ratio.

The association between myosteatosis and adverse outcomes aligns with previous studies across different clinical settings. Loijaard et al., in a retrospective study involving 491 critically ill patients undergoing mechanical ventilation, found that skeletal muscle density and intermuscular adipose tissue were independently associated with 6-month mortality and length of hospital stay [26]. Di Cola et al. reported that, in people living with liver cirrhosis, myosteatosis was a predictor of adverse outcomes and, interestingly it may occur with or without concomitant sarcopenia [6].

Evidence supporting a possible impact of myosteatosis on adverse clinical outcomes is also provided by population-based studies. A longitudinal analysis of the UK Biobank, involving 39,804 participants, showed that adverse muscle composition—including myosteatosis detected by MRI—was a strong predictor of all-cause mortality, with an effect size comparable to that of smoking or a history of cancer [27]. Furthermore, Nachit et al. found that myosteatosis—identified using an artificial intelligence–based analysis of low-dose abdominal CT scans in asymptomatic outpatients—remained independently associated with mortality after multiple adjustments [28].

Although the relevant role of myosteatosis is included in consensus documents regarding sarcopenia [29], dedicated international guidelines for diagnosis, implications, and management of myosteatosis are still an unmet clinical need.

The mechanisms through which myosteatosis may exert detrimental effects are multiple. Fat infiltration into skeletal muscle could lead to dysregulation of muscle endocrine function, promoting lipotoxicity, insulin resistance, and chronic inflammation [9]. This process may contribute to the development of metabolic frailty, as myosteatosis is not only a fat distribution abnormality but also promotes chronic signalling pathways that drive muscle catabolism [5]. Although diabetes may influence ectopic fat deposition, including myosteatosis [30], in our cohort the association between in-hospital mortality and myosteatosis did not differ between people with and without diabetes. This could be due to limited statistical power or could suggest that myosteatosis may exert a detrimental effect or, alternatively, could be a marker of unfavourable metabolic derangement, independently of the underlying mechanism of ectopic fat infiltration.

In our cohort, participants with a higher degree of myosteatosis exhibited higher CONUT scores, indicating a higher risk of malnutrition. Furthermore, after multiple adjustments, the CONUT score was significantly associated with a higher MM/F ratio. These findings are consistent with the study by Donnelly et al., which showed that myosteatosis was associated with biomarkers of poor nutritional status, such as lower total protein and albumin levels, in a population of individuals with inflammatory bowel disease [31]. Similarly, Kusunoki et al. found that intramuscular fat content was a good indicator of risk of poor nutritional status assessed with the PNI index in a cohort of 1,085 individuals with gastrointestinal malignancies [32].

The relationship between the CONUT score and myosteatosis is clinically relevant, as the assessment of myosteatosis relies on advanced imaging techniques that cannot be routinely used for screening unless imaging is performed for other clinical reasons. Patients admitted to internal medicine departments have a relatively high prevalence of malnutrition, and many of them require tailored nutritional therapy and a multidisciplinary approach [20]. To properly address each patient’s nutritional needs, a simple, objective score with a good predictive value should be found. Thus, the definition of easy and cost-effective screening indexes for myosteatosis may be clinically relevant. If further validated, the CONUT score could represent a promising marker of myosteatosis, and its combination with imaging techniques, when feasible, could help to provide a more comprehensive assessment of the nutritional and muscle status of hospitalized patients.

Moreover, the CONUT score has been associated with other measures indicative of unfavourable fat distribution. In fact, Lo Buglio et al. found that hospitalized elderly patients with higher CONUT scores exhibited lower muscle mass and fat-free mass, along with a higher percentage of fat mass [33]. Similarly, a previous study from our group highlighted an association between the CONUT score and liver fat accumulation, as determined by computed tomography [21]. Notably, the CONUT score and myosteatosis showed a significant interaction with kidney transplant–related outcomes [34] possibly indicating an interplay of malnutrition and myosteatosis. Based on this body of evidence, it seems that CONUT score could capture alteration of fat distribution that could impact on clinical outcomes and could have a complementary role to imaging-derived measures.

This study has several strengths. Our study is the first to our knowledge to investigate the short-term prognostic value of the MM/F score in an Internal Medicine setting. Moreover, we aimed to assess the association of CONUT score with CT-measured myosteatosis on admission as a candidate useful index that may enable clinicians to identify patients at risk for worse outcomes. However, there are several limitations regarding this study. There are unmeasured confounders influencing the in-hospital prognosis and myosteatosis even though we performed adjustment for confounders and sensitivity analyses to enhance the robustness of the findings. Information about glycaemic control and diabetes therapy that may have influenced the risk of myosteatosis was not available, although no statistically significant effect modification by type 2 diabetes was detected on the association between myosteatosis and in-hospital mortality. The CONUT score is based on measures that could be affected by acute inflammatory status; however, the score was widely validated in in-hospital settings including surgery, oncology, and internal medicine and we performed a sensitivity analysis including hs-CRP. Furthermore, although a few patients received nutritional evaluation and counselling from the Nutrition Support Team during hospitalization, we are unable to make any inferences regarding the impact of these interventions on in-hospital outcomes or long-term prognosis. This was due to the lack of information on muscular tomodensitometric profile, nutritional status, and adherence after discharge, as well as on the distribution of these interventions across study groups. However, given the observational nature of this study, nutritional interventions were not guided by CONUT score and/or MM/F ratio assessment, but rather by the clinical conditions of the participants, and did not differ from the usual care provided in our Department. The CT scans were not performed with the main aim of measuring MM/F, but for individualized clinical reasons. However, we employed a standardised protocol to measure MM/F to assure maximum reproducibility.

## Conclusions

In this cohort of patients admitted to an Internal Medicine Department, greater myosteatosis, assessed using the MM/F ratio, was associated with higher in-hospital mortality and a higher CONUT score. No statistically significant effect modification by type 2 diabetes was detected, although the analysis may have had limited power to identify subgroup differences. These findings support further investigation of CONUT as a potential screening tool for myosteatosis, but dedicated diagnostic-performance and validation studies are required before its clinical use can be recommended.

## Supplementary Information

Below is the link to the electronic supplementary material.

Supplementary Material 1

## Data Availability

The data that support the findings of this study are available on request from the corresponding author. The data are not publicly available due to privacy or ethical restrictions.
