# Supplementary material for: Myosteatosis is associated with adverse in-hospital outcomes and CONUT score in hospitalized internal medicine patients
Source: Acta Diabetol. 2026 Aug 31;63(12):2107–16. doi: 10.1007/s00592-026-02785-3 (PMC13769740; doi:10.1007/s00592-026-02785-3)
Supplement: Supplementary file 1 [file 592_2026_2785_MOESM1_ESM.docx]

**Table S1. Logistic regression analyses assessing the association of in-hospital death with MM/F ratio**

|  | **Model 1** | | | **Model 2** | | |
| --- | --- | --- | --- | --- | --- | --- |
| Covariates | OR | 95%CI | *P* | OR | 95%CI | *P* |
| **MM/F ratio** | 2.45 | 1.07 – 6.21 | **0.041** | 2.74 | 1.11 – 7.75 | **0.040** |
| **Age, years** | 1.01 | 0.99 – 1.05 | 0.376 | 1.00 | 0.96 – 1.04 | 0.942 |
| **Male sex** | 0.69 | 0.32 – 1.50 | 0.350 | 0.47 | 0.19 – 1.10 | 0.084 |
| **Type 2 diabetes** |  |  |  | 0.58 | 0.22 – 1.42 | 0.247 |
| **eGFR, ml/min/1.73m^2^** |  |  |  | 0.99 | 0.97 – 1.00 | 0.063 |
| **Active malignancies** |  |  |  | 8.39 | 3.46 – 22.45 | **<0.001** |
| **Sepsis** |  |  |  | 2.43 | 1.02 – 5.94 | **0.046** |

Odds ratios (HRs) and 95% confidence intervals (95%CI) as estimated with logistic regression analyses with in-hospital death as dependent variable and myosteatosis assessed as multifidus muscle to subcutaneous fat (MM/F) ratio as independent variable.

Model 1: adjusted for age and sex

Model 2: Model 1 + history of type 2 diabetes, estimated glomerular filtration rate (eGFR), active malignancies, and sepsis during in-hospital stay.

**Table S2. Penalized Firth’s logistic regression analysis assessing the association of in-hospital death with MM/F ratio**

|  | **Firths’ logistic regression** | | |
| --- | --- | --- | --- |
| Covariates | OR | 95%CI | *P* |
| **MM/F ratio** | 2.49 | 1.04 – 5.96 | **0.032** |
| **Age, years** | 1.00 | 0.96 – 1.04 | 0.999 |
| **Male sex** | 0.48 | 0.21 – 1.08 | 0.068 |
| **Type 2 diabetes** | 0.61 | 0.25 – 1.45 | 0.258 |
| **eGFR, ml/min/1.73m^2^** | 0.99 | 0.97 – 1.00 | 0.068 |
| **Active malignancies** | 7.27 | 3.06 – 17.30 | **<0.001** |
| **Sepsis** | 2.43 | 1.02 – 5.94 | **0.046** |

Odds ratios (HRs) and 95% confidence intervals (95%CI) as estimated with Firth’s logistic regression analysis with in-hospital death as dependent variable and myosteatosis assessed as multifidus muscle to subcutaneous fat (MM/F) ratio as independent variable. The model was adjusted for age, sex, history of type 2 diabetes, estimated glomerular filtration rate (eGFR), malignancies, and sepsis during in-hospital stay.

**Table S3. Multiple regression analysis assessing the major determinants of MM/F ratio.**

|  | **MM/F ratio** | | |
| --- | --- | --- | --- |
| Covariates | β | 95%CI | *P* |
| **Age, years** | 0.48 | 0.31 – 0.66 | **<0.001** |
| **Male sex** | -0.20 | -0.35 – -0.06 | **0.006** |
| **Being bedridden** | 0.04 | -0.14 – 0.22 | 0.647 |
| **Active malignancies** | 0.02 | -0.13 – 0.16 | 0.837 |
| **Type 2 diabetes** | -0.04 | -0.19 – 0.10 | 0.554 |
| **CHD** | -0.02 | -0.17 – 0.13 | 0.817 |
| **Stroke/TIA** | -0.05 | -0.19 – 0.09 | 0.464 |
| **CKD** | -0.03 | -0.18 – 0.12 | 0.681 |
| **Dementia** | 0.12 | -0.05 – 0.28 | 0.177 |
| **COPD** | 0.06 | -0.09 – 0.21 | 0.407 |
| **CONUT score** | 0.30 | 0.14 – 0.47 | **<0.001** |

Standardized β coefficients and the 95% confidence intervals as estimated by multiple regression analysis with multifidus muscle to subcutaneous fat (MM/F) ratio as dependent variable and age, sex, being bedridden, active malignancies, history of diabetes, coronary heart disease (CHD), stroke/transient ischemic attack (TIA), chronic kidney disease (CKD), dementia, chronic obstructive pulmonary disease (COPD) and controlling nutritional status (CONUT) score as explanatory variables. ***P* < 0.01, *** *P* < 0.001. Blue: β ≥ 1.00, red: β < 1.00.
